# Modeling study of a Central Pattern Generator in the Melibe seaslug

**DOI:** 10.1186/1471-2202-13-S1-P187

**Published:** 2012-07-16

**Authors:** Sajiya Jalil, Dane Allen, Andrey Shilnikov

**Affiliations:** 1 Department of Mathematics & Statistics, Georgia State University, Atlanta, GA 30303, USA; 2 Neuroscience Institute, Georgia State University, Atlanta, GA, 30303, USA

## 

Behaving animals show correlated neuronal activities in their nervous system. These observations lead to the question, how are the specific patterns of activities generated and used to control behaviors? Central Pattern Generators (CPGs) are small networks of neurons that are experimentally identified as necessary for expressions of behaviors, and the core group capable of controlling various aspects of the behavior. Mechanistic understanding of CPG functions is under intense investigation [1]. In this study, we investigate the role of network configurations of an experimentally based CPG for swimming behavior in the marine invertebrate Melibe leonine [2].

Repetitive nature of activity patterns of CPGs can be examined through the relative phase relations between bursting interneurons. Experimentally and computationally, such phase-lags are measured with respect to a reference neuron, thus allowing for an analysis of various activity patterns in a systematic way. For the Melibe CPG model under consideration, we consider four Hodgkin-Huxley type square-wave bursters, connected via fast non-delayed inhibitory synapses represented by fast threshold modulating function. Due to intrinsic symmetry, the network can be treated as two pairs of half center oscillators (HCOs). In a HCO both neurons reciprocally inhibit each other, leading to alternate, anti-phase bursting patterns, which can coexist with in-phase bursting when the HCO is inhibited externally [3,4]. In the given model, one HCO inhibits the other one and gets a positive feedback in the form of excitatory drive. We will further enhance the CPG model by including additional interneurons to reflect physiological fine details observed in-vitro. The exploration of stable phase-locked states, perturbed externally by applied current pulses, allows us to suggest plausible mechanisms of the CPG and come up with predictions that may be verified by electrophysiological experiments.

The phase-locked pattern, observed in the experiment, is identified as an attractor in numerical simulations. We find that specific distributions of synaptic conductance shift the attractor so that it merges with a saddle corresponding to unstable phase-locked state. Investigation indicates that weak excitatory connections play no significant role in the generation of the pattern but may have stabilizing effect. Electrical coupling, which appears to be very weak and negligible in the physiological context, may play a critical role in altering the behavior of the network.

## Conclusions

We conclude that the idiosyncratic phase-locking seen in the experimental setup is due to a specific network configuration of the CPG model where a driving HCO uni-directionally inhibits a nearly identical one, provided that coupling strengths stay rather weak. Future investigations will be focused on enhanced realistic CPG models with additional connections and participating interneurons.
